# Clinical trial participant information leaflets overstate the harms/risks of participation and understate the benefits

**DOI:** 10.1371/journal.pone.0346984

**Published:** 2026-05-05

**Authors:** Rafaela Carapeto, Andrew Willis, Frances Shiely

**Affiliations:** 1 Trials Research and Methodologies Unit, HRB Clinical Research Facility, University College Cork, Cork, Ireland; 2 Health Service Executive, Cork, Ireland; 3 School of Public Health, University College Cork, Cork, Ireland; Johns Hopkins: Johns Hopkins University, UNITED STATES OF AMERICA

## Abstract

**Objectives:**

Participant Information Leaflets (PILs) are essential for informed consent in randomised controlled trials (RCTs), detailing the trial’s purpose, methods, and potential benefits and harms/risks to aid participant decision-making. The purpose of our study was to establish the balance of benefits and harms/risks communicated to trial participants in PILs.

**Study Design and Setting:**

A retrospective study of 228 PILs from trials in the UK and Ireland. Benefits and harms/risks were defined and quantified through a systematic extraction and coding process. The analysis included the calculation of mean, median, and ranges for word counts of positive and negative reinforcement statements, and their proportional representation in the overall content of the PILs.

**Results:**

The study included PILs from 26 Clinical Trial Units and Clinical Research Facilities, covering 228 unique studies in the UK and Ireland. The word count for positive reinforcement statements (median: 56 words, 2.3% of total PIL word count) was significantly lower than for negative reinforcement statements (median: 149 words, 5.7% of total PIL word count). Approximately 72% of PILs contained a higher percentage of words dedicated to negative rather than positive reinforcement, indicating an imbalance in presenting harms/risks over benefits.

**Conclusions:**

The findings suggest that information in participant information leaflets places greater emphasis on potential harms and risks than on potential benefits of trial participation. While regulatory requirements necessitate clear communication of possible harms, the relatively limited emphasis on potential benefits may influence how participants interpret trial information. These findings highlight the importance of ensuring that participant-facing materials present both potential harms and benefits in a balanced and accessible way to support informed decision-making. Greater guidance for trial teams on how to present this information may help improve the clarity and balance of participant information leaflets.

## Background

Prior to participation in a randomised controlled trial (RCT), potential participants, or their representatives, are provided with a participant information leaflet (PIL) as mandated by the Clinical Trials – Regulation EU No 536/2014 of the European Parliament [1]. A PIL provides all information on the trial that participants are being invited to take part in, so they can make a decision on participation, or not [2,3]. This is called providing informed consent, which is a regulatory requirement [1,4]. Essential information includes: the trial purpose, the methods of data collection, potential risks, benefits, as well as freedom to withdraw from the study at any time without any impact on current treatment [3].

Participants’ response to the PIL can significantly affect recruitment. Historically, the design of PILs has been tailored for the purposes of documenting the consent process, not participant comprehension [5]. PILs have also been criticised for being unnecessarily complex and long [2,3]. Accurate comprehension of information within the PIL pertaining to the risks and benefits of trial participation is essential in informing decision making by trial participants [6]. Presentation of this information within PILs has further consequences when shaping participants’ expectations and experiences within a trial, which, if met, can improve participant response to treatment [7], and if not met, may lead to drop outs, dissuade future participation in research [8], or potentially lead to a ‘nocebo’ effect (where negative expectations regarding an intervention can lead to more adverse outcomes within a trial) [9,10]. Ensuring accuracy and balance in the presentation of information relating to the advantages and disadvantages of trial participation is important in facilitating informed decision making and providing an accurate, unbiased description of the intervention being tested [6].

Standardised PILs do not exist, therefore trial teams create bespoke documents for their own trial. There is significant variability in layout, information provided, and length, thus uncertainty remains about the information trialists should communicate to potential trial participants to improve recruitment [11–13] and retention [14–16]. The PRioRiTy study, conducted in a priority setting partnership, ranked it as second out of twenty priorities in terms of importance [17]. While the Clinical Trials Regulation mandates that the nature, objectives, benefits, implications, harms/risks and inconveniences of the clinical trial are communicated to potential trial participants when taking informed consent [18–20], it is not clear if the benefits and risks are equally communicated.

The purpose of this study was to evaluate the balance in information attributed to the benefits and harms/risks in PILs used for trial recruitment. While regulatory frameworks emphasise the need to clearly communicate potential harms and risks, there is limited empirical evidence examining how this information is balanced against potential benefits in practice. By systematically quantifying the emphasis placed on benefits and harms/risks in PILs, this study addresses an important gap in the literature and provides evidence to inform future guidance on the development of participant-facing trial information.

## Methods

### Study design

This was a retrospective, cross-sectional analysis of PILs used to recruit participants to RCTs in the United Kingdom and Ireland. The study examined the content of PILs to assess the balance of information relating to potential benefits and harms/risks of trial participation.

### Data collection

In a previous study we collected a corpus of PILs(3). In brief, we contacted Clinical Trial Units/Clinical Research Facilities in Ireland and the United Kingdom (UK) and asked them to provide us with PILs and/or Informed Consent Forms (ICFs) and other available materials used to recruit participants to RCTs. These included trials within cohorts (TWiCs), feasibility and pilot studies. Some trial teams agreed to share the PILs/ICFs as open access, and the original study team has made them available on the Trial Forge Website (https://www.trialforge.org/excelsior-pil-library/). The PILs include; PILs written for adult participants, PILs provided to parents whose children were being recruited to a trial, PILs for legal representatives/family members of patients who did not have capacity to consent, PILs for adults who regained capacity during a trial, and PILs written for children. Our sample included PILs from 21 CTUs in England, Scotland, and Wales (of 34 registered in the UK and Northern Ireland) and all 5 of the CRFs operating at the time of data collection in the Republic of Ireland.

This was a retrospective study of PILs, with no human subject contact, therefore ethical approval for the study was not required.

### Data extraction

For our study, we were interested in assessing the balance of the information provided in the PILs on benefits and harms/risks. We defined benefits as any “positive reinforcement statement”, i.e., any statement on the benefits of participation in the trial or one that encouraged the participants to partake or stay in the trial. We defined harms/risks as any “negative reinforcement statement”, i.e., any statement on the harms/risks of trial participation, the right to withdraw or any statement that was associated with non-participation. We extracted all positive reinforcement statements and all negative reinforcement statements, as well as descriptive information on the trials. All extracted information was recorded in MS Excel.

### Coding

**Fig 1:**
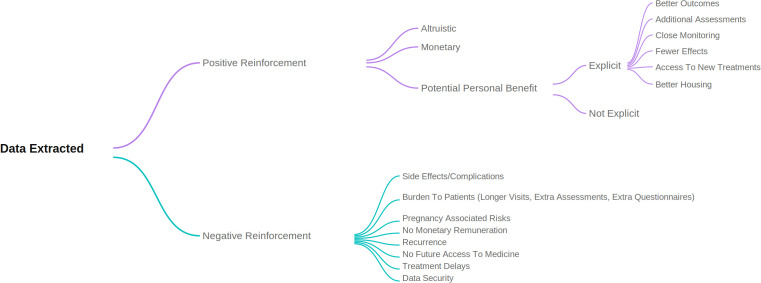
Coding framework.

RC and FS met five times over the course of seven weeks, to develop and refine the coding framework used to classify statements relating to potential benefits and harms/risk in the PILs. RC conducted the initial coding of all statements, while FS independently reviewed the coding framework and verified the categorisation of statements. This iterative process continued until no new categories or terms were identified in the PILs. Where uncertainty arose regarding the categorisation of statements, these were discussed between RC and FS until agreement was reached. FS also conducted confirmatory coding on a random 10% sample of the PILs to ensure consistency in the application of the coding framework. A summary of the final coding framework is presented in Fig 1.

#### *Positive reinforcement statements (categories summarised in* Fig 1).

Text that stated that participating in the trial could benefit others or the disease under trial was coded as ‘Altruistic’. If monetary incentives were given it was coded as ‘Monetary’. The potential personal benefit that was explicit to the participants was coded according to the type of benefit – ‘additional assessments’, ‘better outcomes’, ‘fewer side effects’, ‘close monitoring’, ‘access to newer treatments’ and ‘better housing’. In these scenarios the PIL authors clearly stated how participants would benefit from joining the clinical trial in the management of their condition. If the word benefit was mentioned but not explained, then it was coded as ‘Potential personal benefit-not explicit’.

#### *Negative reinforcements statements (categories summarised in* Fig 1).

Lists of side effects from drugs/procedures or potential complications from the intervention were coded as ‘Side effects/Complications’. This included surgical procedures, use of medications, radiotherapy and psychological side-effects. Any type of attendance outside standard of care, increased waiting times and/or questionnaires that wouldn’t routinely be given to patients were coded as ‘Burden to the participants’. In some PILs this included psychological burden directly attributed to increased attendance. Any type of written text that clearly indicated women should not get pregnant while participating in the trial, or that contraception needed to be used while participating in the trial to prevent any harm to the unborn foetus, was coded as ‘pregnancy associated risks’. In some PILs this included psychological burden. Any expenses incurred by the participants while taking part, e.g., costs of travel, parking and food, or explicit statements about participants not receiving any monetary incentive, were coded as ‘non-monetary remuneration’. Text that stated that because of taking part the participants condition worsened, and they would have to receive extra treatment, was coded as ‘recurrence’. If the participant was told they would no longer have access to the medicine once the trial was completed, it was coded as ‘no future access to the medicine’. If the patient would have to wait days, weeks or months for the intervention, it was coded as ‘treatment delays’. Text on data security breaches were coded as ‘data security’.

### Data analysis

We calculated the mean (standard deviation), median (interquartile range), minimum and maximum word count for positive and negative reinforcements per PIL. We also did the same for the overall PIL word count.

## Results

Twenty-six CTUs/CRFs provided Patient Information Leaflets relating to 228 unique studies which were included in our analysis. Of these 228 studies, 227 (99.6%) were randomised controlled designs with 2 or more arms, one trial (0.4%) was a multifactorial platform design.

Randomisation was conducted at the individual participant level for 97.8% (n = 223) of trials and at the cluster level in 2.2% (n = 5) of trials. Funding information was included in 204/228 included PILs. Of the 204 included study PILs with funding information, 24 (11.8%) specified commercial sponsorship, 180 (88.2%) study PILs had a non-commercial sponsor.

Table 1 provides a descriptive summary of the word count in the PILs. Word count for PILs ranged from 53 to 11,489, with a median 2596 (IQR 1892). For positive reinforcement statements, word count ranged from 0 to 534 (median 56 (IQR 51)). The median proportion of total PIL word count attributable to positive reinforcement was 2.36% IQR (2.17%), Word count for negative reinforcement statements ranged from 0 to 2906 words (median 149; IQR 48–321). The median proportion attributable to negative reinforcement was 5.57% (IQR 6.92%)

**Table 1:** PIL Word count.

N = 228	Total Word Count	Positive Reinforcement Word Count	Negative Reinforcement Word Count
**Mean (SD)**	2994.77 (1996.01)	73.03 (58.47)	292(485.23)
**Median (IQR)**	2605 (1746-3573)	56 (40-91)	149 (48-321)
**Max**	11489	534	2906
**Min**	53	0	0

Almost three-quarters of PILs (72%; 164/228) had a greater percentage of negative reinforcement words, compared to positive reinforcement words. Six PILs had no positive reinforcement words at all. Of those six, two had no negative reinforcement words either. Both were children PILs. Table 2 shows the number (%) of PILs categorised by the percentage of total word count devoted to positive and negative reinforcement. At lower percentage levels, positive reinforcement generally exceeds negative reinforcement, with the exception of the < 1% category.. However, as the proportion of reinforcement-related content increases, negative reinforcement becomes progressively more common. This trend is reflected in the ratios, which are greater than one in most lower and mid-range categories, but fall below one in the highest category.

**Table 2:** No. of PILs by percentage of total word count devoted to positive and negative reinforcement (n = 228).

Percentage of total word count	Positive reinforcement, N (%)	Negative reinforcement, N (%)	Ratio (positive:negative)
**<1**	19 (8)	37 (16)	0.5
**1-1.9**	69 (30)	10 (4)	6.9
**2.0-2.9**	51 (22)	18 (8)	2.8
**3.0-3.9**	45 (20)	28 (12)	1.6
**4.0-4.9**	12 (5)	13 (6)	0.9
**5.0-5.9**	15 (7)	15 (7)	1.0
**>6**	17 (7)	107 (47)	0.2

### Positive reinforcement

We identified 222 PILs (97.4%) that contained positive reinforcement statements. The most common form of positive reinforcement related to altruistic benefits (Table 3), with 93.2% (n = 207) indicating that trial participation could improve understanding of the disease or benefit future patients. Potential personal benefit was reported in 30.6% of PILs. However, 14.1% (n = 32) did not explain to participants how they could potentially benefit from taking part in the trial. Financial travel incentives (monetary) were the third most common form of positive reinforcement (13.1%, n = 29).

**Table 3:** Number (%) of participant information leaflets containing each category of positive reinforcement (n = 228).

Positive Reinforcement	Total number of PILs	%
Altruistic	207	93.2%
Potential personal benefit, explicit – better outcomes	68	30.6%
Monetary	29	13.1%
Potential personal benefit, explicit – additional assessments	28	12.6%
Potential personal benefit, explicit – close monitoring	9	4.1%
Potential personal benefit, explicit – fewer side effects	5	2.3%
Potential personal benefit, explicit – access to new treatments	5	2.3%
Potential personal benefit, explicit -better housing	1	0.5%
Potential personal benefit, not explicit	32	14.1%

* The total adds to more than 228 because some PILs had negative reinforcement statements in multiple categories.

### Negative Reinforcement

Negative reinforcement statements were identified in 203 out of 228 PILs (89%). Twenty-five PILs (11%) contained no negative reinforcement, the majority of which were children’s PILs that didn’t include detailed descriptions of potential side effects or risks. Two additional PILs were for adults who had regained capacity. As expected, due to regulatory requirements, the most frequent type of negative reinforcement described in the PILs was side effects/complications from the intervention (Table 4).

**Table 4:** Number (%) of participant information leaflets containing each category of negative reinforcement.

Discourage/Risk	N*	%
Side effects/Complications	176	87.1%
Burden to patient (longer visits, extra assessments, extra questionnaires, etc)	50	24.8%
Pregnancy associated risks	18	9.1%
Psychological	16	8.9%
No monetary remuneration	8	4.0%
Risk of recurrence	7	3.5%
No future access to medicine	4	2.0%
Treatment delays	2	1.0%
Data security	1	0.5%

* The total adds to more than 228 because some PILs had negative reinforcement statements in multiple categories.

## Discussion

The purpose of this study was to evaluate the balance of information relating to potential benefits and harms/risks presented to participants in PILs used for trial recruitment. Our analysis of 228 PILs from trials conducted in the United Kingdom and Ireland demonstrates a clear imbalance, with substantially more emphasis placed on harms/risks than on potential benefits of participation. In particular, the median word count devoted to negative reinforcement statements was almost three times greater than that devoted to positive reinforcement statements, suggesting that participant-facing trial information may disproportionately foreground risks over benefits.

We know that participants often take part in trials hoping to achieve better outcomes [21] while also being motivated by altruistic reasons [22,23]. However, the PILs analysed in this study emphasised altruistic benefits far more frequently than potential personal benefits. While 93.2% of PILs highlighted the potential contribution of the trial to improving understanding of the disease or benefiting future patients, fewer than one-third (30.6%) included information about potential personal health benefits. This imbalance represents a missed opportunity for trial teams and could influence participants’ expectations when considering participation. Underrepresentation of potential personal benefit may direct attention towards potential harms/risks associated with participation rather than possible direct health benefits. The framing of information in this way may also have implications for the nocebo effect, whereby negative expectations about an intervention can influence the experience or reporting of adverse outcomes. When PILs emphasise potential harms or side effects more strongly than potential benefits, they may inadvertently shape participants’ expectations in ways that heighten awareness of negative outcomes. Previous research suggests that expectation setting can influence both perceptions of treatment and reported side effects [7]. While it is ethically and legally necessary to clearly communicate potential harms, presenting this information in a disproportionately negative way may unintentionally contribute to expectation-related effects. Ensuring a more balanced presentation of harms/risks and benefits may therefore support informed decision-making and should be considered by trial teams.

The predominance of negative reinforcement in PILs, accounting for approximately 5.7% of the total word count compared to only 2.3% for positive reinforcements, suggests a cautious approach potentially aimed at over-protecting participants from trial harms/risks, acknowledging the fact that it is a regulatory requirement to notify participants of potential risks or harms. We suggest a more balanced approach to providing information on both harms and benefits so that potential trial participants are not unnecessarily discouraged from trial participation. We also recommend making the participant information leaflet more readable in terms of its length and complexity, which has been critiqued previously in relation to their contribution to misunderstandings about the trials’ risks and benefits [2,24], and suitable for a lay audience (those we aim to recruit) which research has shown even trial lay summaries are not suitable for [25].

The variability in PIL content, reflected in the wide range in word counts and differences on the types of information emphasised, suggests a lack of standardisation in how trial information is presented to potential participants. This is concerning given the central role of these documents in supporting informed consent. In line with the Declaration of Helsinki, investigators have a responsibility to ensure that research participants are sufficiently informed to enable them to provide valid consent [26]. While regulatory guidance exists on the information that should be included in PILs, there is limited direction on how benefits and harms should be balanced in their presentation. Recent guidance developed through an expert consensus process provides recommendations to support trial teams in preparing accessible and understandable PILs and consent forms [27]; however, the balance between communicating harms and potential benefits is not explicitly addressed.

From a practical perspective, trial teams and regulatory bodies could consider introducing clearer guidance on how information about benefits and harms/risks should be presented within participant information leaflets. For example, templates could include dedicated sections describing both the potential benefits and the potential harms of participation, ensuring that each is clearly addressed rather than dispersed throughout the document. Guidance could also encourage trial teams to describe potential personal benefits where appropriate, alongside commonly stated altruistic benefits. While it may not be appropriate to mandate strict ratios of text devoted to harms and benefits, encouraging trial teams to review the relative prominence of these sections during PIL development may help promote a more balanced presentation of information. Ethics committees and regulatory bodies reviewing trial documentation could also consider whether both benefits and harms are communicated clearly and proportionately as part of their assessment of participant-facing materials. Such approaches may contribute to improving participant understanding and supporting informed decision-making.

Interpretation of information on harms/risks and benefits in participant information leaflets is influenced not only by the amount of text devoted to each, but also by how that information is presented. Brief statements describing serious harms or side effects, such as death or serious injury, are likely to have a greater impact on participation decisions than longer explanations describing potential future benefits. Qualitative research is needed to delve deeper into this issue. However, what we do know is that we need to ensure decisions regarding participation are fully informed by comprehensible and balanced information on benefits and harms/risks [9]. Previous research has also highlighted the importance of co-developing recruitment materials with user groups who are representative of the target patient population [28].

### Strengths and limitations

This study included PILs from a range of different CTUs which were geographically diverse and covered almost all areas of the UK. Included PILs covered different disease areas and were targeted towards diverse participant population, including adults children and those with impaired capacity to consent. Our findings therefore have broad applicability and are not limited to specific clinical populations or trial types.

This study exclusively examined the PIL and the text concerning benefits and harms/risks contained within it. We did not take into account the discussions between recruiters and potential trial participants during the informed consent process. One limitation of the analysis is the use of word count as a measure of emphasis, which may represent an over-simplification of how individuals interpret information when deciding whether to participate in a trial. A short, simple statement describing a serious harm or treatment side effect, such as death or serious injury, is likely to have a greater impact on decision-making than a longer explanation describing potential future benefits. Future research could build on this work by incorporating qualitative approaches to explore how potential participants interpret and respond to information about harms and benefits in PILs. Methods such as interviews or focus groups could help assess how individuals perceive and prioritise different types of statements, and how the framing of information influences expectations and decision-making. Combining quantitative assessments of information balance with qualitative insights into participant interpretation would provide a more comprehensive understanding of how trial information materials influence recruitment and informed consent.

## Conclusion

This study highlights an imbalance in the weighting of information on the benefits and harms/risks provided to potential trial participants in Participation Information Leaflets. The trial community acknowledges the need to make trial participants aware of the potential harm/risk of trial participation, but equally, informing them of the benefits should have equal prominence. Standardising this in PILs is needed to ensure that participants are provided with accurate, balanced information pertaining to benefits and harms/risk, in formats that are easy to comprehend, so that they can make an informed decision regarding participation.

**Key findings**

- Comprehensive analysis of 228 PILs reveals a disproportionate focus on negative over positive reinforcements, quantifying the extent of imbalance in trial information leaflets.
- Empirical evidence suggests that the median word count for harms/risks significantly exceeds that for benefits, with potential implications for participant perception and decision-making.
- The study underscores the need for regulatory and practice changes to ensure balanced information about both the benefits and harms/risks of trial participation, fostering more informed consent.

## References

[1] Petrini C. Regulation (EU) No 536/2014 on clinical trials on medicinal products for human use: an overview. Ann Ist Super Sanita. 2014;50(4):317–21. doi: 10.4415/ANN_14_04_04 25522070

[2] O’Sullivan L, Sukumar P, Crowley R, McAuliffe E, Doran P. Readability and understandability of clinical research patient information leaflets and consent forms in Ireland and the UK: a retrospective quantitative analysis. BMJ Open. 2020;10(9):e037994. doi: 10.1136/bmjopen-2020-037994 32883734 PMC7473620

[3] Shiely F, Murphy E, Gilles K, Hood K, O’Sullivan L, Harman N, et al. Trial participants’ self-reported understanding of randomisation phrases in participation information leaflets can be high, but acceptability of some descriptions is low, especially those linked to gambling and luck. Trials. 2024;25(1):391. doi: 10.1186/s13063-024-08217-3 38890748 PMC11186130

[4] International Conference on Harmonisation. ICH Harmonised Guideline Integrated Addendum to ICH E6(R1): Guideline for Good Clinical Practice E6(R2). 2016. http://www.ich.org/products/guidelines/efficacy/efficacy-single/article/integrated-addendum-good-clinical-practice.html

[5] Simonds VW, Garroutte EM, Buchwald D. Health Literacy and Informed Consent Materials: Designed for Documentation, Not Comprehension of Health Research. J Health Commun. 2017;22(8):682–91. doi: 10.1080/10810730.2017.1341565 28759329 PMC6155979

[6] Cuddihy L, Howick J, Murphy E, Shiely F. When describing harms and benefits to potential trial participants, participant information leaflets are inadequate. Trials. 2024;25(1):292. doi: 10.1186/s13063-024-08087-9 38693579 PMC11061982

[7] Akroyd A, Gunn KN, Rankin S, Douglas M, Kleinstäuber M, Rief W, et al. Optimizing patient expectations to improve therapeutic response to medical treatment: A randomized controlled trial of iron infusion therapy. Br J Health Psychol. 2020;25(3):639–51. doi: 10.1111/bjhp.12435 32519431

[8] Skea ZC, Newlands R, Gillies K. Exploring non-retention in clinical trials: a meta-ethnographic synthesis of studies reporting participant reasons for drop out. BMJ Open. 2019;9(6):e021959. doi: 10.1136/bmjopen-2018-021959 31164359 PMC6561611

[9] Kirby N, Shepherd V, Howick J, Betteridge S, Hood K. Nocebo effects and participant information leaflets: evaluating information provided on adverse effects in UK clinical trials. Trials. 2020;21(1):658. doi: 10.1186/s13063-020-04591-w 32680561 PMC7368797

[10] Howick J, Webster R, Kirby N, Hood K. Rapid overview of systematic reviews of nocebo effects reported by patients taking placebos in clinical trials. Trials. 2018;19(1):674. doi: 10.1186/s13063-018-3042-4 30526685 PMC6288933

[11] Preston NJ, Farquhar MC, Walshe CE, Stevinson C, Ewing G, Calman LA, et al. Strategies designed to help healthcare professionals to recruit participants to research studies. Cochrane Database Syst Rev. 2016;2(2):MR000036. doi: 10.1002/14651858.MR000036.pub2 35658160 PMC8190980

[12] Murphy E, O’Keeffe A, O Shea N, Long E, Eustace JA, Shiely F. Patient perceptions of the challenges of recruitment to a renal randomised trial registry: a pilot questionnaire-based study. Trials. 2021;22(1):597. doi: 10.1186/s13063-021-05526-9 34488851 PMC8420031

[13] Brady LJL, Yu EY, Lin D, Gore JL, Nelson PS, Shiely F, et al. Examining clinical perspectives and strategies for improving enrolment of underserved communities in prostate cancer clinical trials: an examination of race and ethnicity. Am J Clin Exp Urol. 2023;11(5):385–94. 37941652 PMC10628627

[14] Murphy E, Gillies K, Shiely F. How do trial teams plan for retention during the design stage of the trial? A scoping review. Trials. 2023;24(1):784. doi: 10.1186/s13063-023-07775-2 38049833 PMC10694955

[15] Murphy E, Gillies K, Shiely F. Retention strategies are routinely communicated to potential trial participants but often differ from what was planned in the trial protocol: an analysis of adult participant information leaflets and their corresponding protocols. Trials. 2024;25(1):372. doi: 10.1186/s13063-024-08194-7 38858790 PMC11163762

[16] Murphy E, McCann S, Shiely F, Gillies K. Planning retention strategies in clinical trials-a qualitative interview study with members of trial teams. J Clin Epidemiol. 2025;188:111979. doi: 10.1016/j.jclinepi.2025.111979 40976518

[17] Healy P, Galvin S, Williamson PR, Treweek S, Whiting C, Maeso B, et al. Identifying trial recruitment uncertainties using a James Lind Alliance Priority Setting Partnership - the PRioRiTy (Prioritising Recruitment in Randomised Trials) study. Trials. 2018;19(1):147. doi: 10.1186/s13063-018-2544-4 29490702 PMC5831203

[18] Grady C. Enduring and emerging challenges of informed consent. N Engl J Med. 2015;372(9):855–62. doi: 10.1056/NEJMra1411250 25714163

[19] Gupta UC. Informed consent in clinical research: Revisiting few concepts and areas. Perspect Clin Res. 2013;4(1):26–32. doi: 10.4103/2229-3485.106373 23533976 PMC3601699

[20] European Parliament, Council of the European Union. Regulation (EU) No 536/2014 on clinical trials on medicinal products for human use and repealing Directive 2001/20/EC. 2014.

[21] Locock L, Smith L. Personal benefit, or benefiting others? Deciding whether to take part in clinical trials. Clin Trials. 2011;8(1):85–93. doi: 10.1177/1740774510392257 21163854

[22] Godskesen T, Hansson MG, Nygren P, Nordin K, Kihlbom U. Hope for a cure and altruism are the main motives behind participation in phase 3 clinical cancer trials. Eur J Cancer Care (Engl). 2015;24(1):133–41. doi: 10.1111/ecc.12184 24467443

[23] Sheridan R, Martin-Kerry J, Hudson J, Parker A, Bower P, Knapp P. Why do patients take part in research? An overview of systematic reviews of psychosocial barriers and facilitators. Trials. 2020;21(1):259. doi: 10.1186/s13063-020-4197-3 32164790 PMC7069042

[24] O’ Sullivan L, Feeney L, Crowley RK, Sukumar P, McAuliffe E, Doran P. An evaluation of the process of informed consent: views from research participants and staff. Trials. 2021;22(1):544. doi: 10.1186/s13063-021-05493-1 34407858 PMC8371296

[25] Shiely F, Daly A. Trial lay summaries were not fit for purpose. J Clin Epidemiol. 2023;156:105–12. doi: 10.1016/j.jclinepi.2023.02.023 36868328

[26] World Medical Association. World Medical Association Declaration of Helsinki: ethical principles for medical research involving human subjects. JAMA. 2013;310(20):2191–4. 24141714 10.1001/jama.2013.281053

[27] Coleman E, O’Sullivan L, Crowley R, Hanbidge M, Driver S, Kroll T, et al. Preparing accessible and understandable clinical research participant information leaflets and consent forms: a set of guidelines from an expert consensus conference. Res Involv Engagem. 2021;7(1):31. doi: 10.1186/s40900-021-00265-2 34006326 PMC8130271

[28] Innes K, Cotton S, Campbell MK, Elliott J, Gillies K. Relative importance of informational items in participant information leaflets for trials: a Q-methodology approach. BMJ Open. 2018;8(9):e023303. doi: 10.1136/bmjopen-2018-023303 30185580 PMC6129101

